# Rapid progressive spontaneous hemothorax caused by carcinoma of unknown primary: A very rare case

**DOI:** 10.1002/rcr2.1215

**Published:** 2023-09-12

**Authors:** Sachie Koike, Nobutaka Kobayashi, Masahisa Miyazawa, Naho Yamashita

**Affiliations:** ^1^ Department of Thoracic Surgery Japanese Red Cross Society Nagano hospital Nagano Japan; ^2^ Division of General Thoracic Surgery, Department of Surgery Shinshu University School of Medicine Matsumoto Japan; ^3^ Department of pathology Japanese Red Cross Society Nagano hospital Nagano Japan

**Keywords:** carcinoma of unknown primary, disseminated intravascular coagulation, spontaneous hemothorax, surgical treatment for hemothorax, very rare case

## Abstract

We present a very rare case of spontaneous hemothorax which was caused by carcinoma of unknown primary. To the best of our knowledge, there are no other such cases reported worldwide. The patient was 72‐year‐old male who was referred to our department for massive hemothorax. We undertook surgical treatment for haemostasis, and found multiple tumours which led to the diagnosis of carcinoma of unknown primary as the cause of haemorrhage. The tumours increased and grew rapidly after surgery and hemothorax progressed despite our treatment. The patient died from disseminated intravascular coagulation caused by continuous bleeding on postoperative day 19.

## Graphical abstract

We present a case of spontaneous hemothorax which was caused by carcinoma of unknown primary. Since it was very rare cause of hemothorax, we had difficulty in deciding the course of treatment.

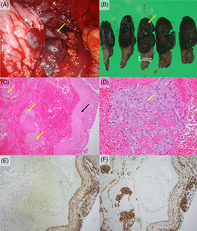

## INTRODUCTION

Hemothorax is mostly caused by trauma or iatrogenic, and spontaneous hemothorax is much less common.^1^ Spontaneous hemothorax is mainly caused by pneumothorax, coagulopathy, vascular disease, neoplasm, and miscellaneous.^1^ We present a case of spontaneous hemothorax which was caused by carcinoma of unknown primary. Since it was a very rare cause of hemothorax, we had difficulty in deciding the course of treatment.

## CASE REPORT

**FIGURE 1:**
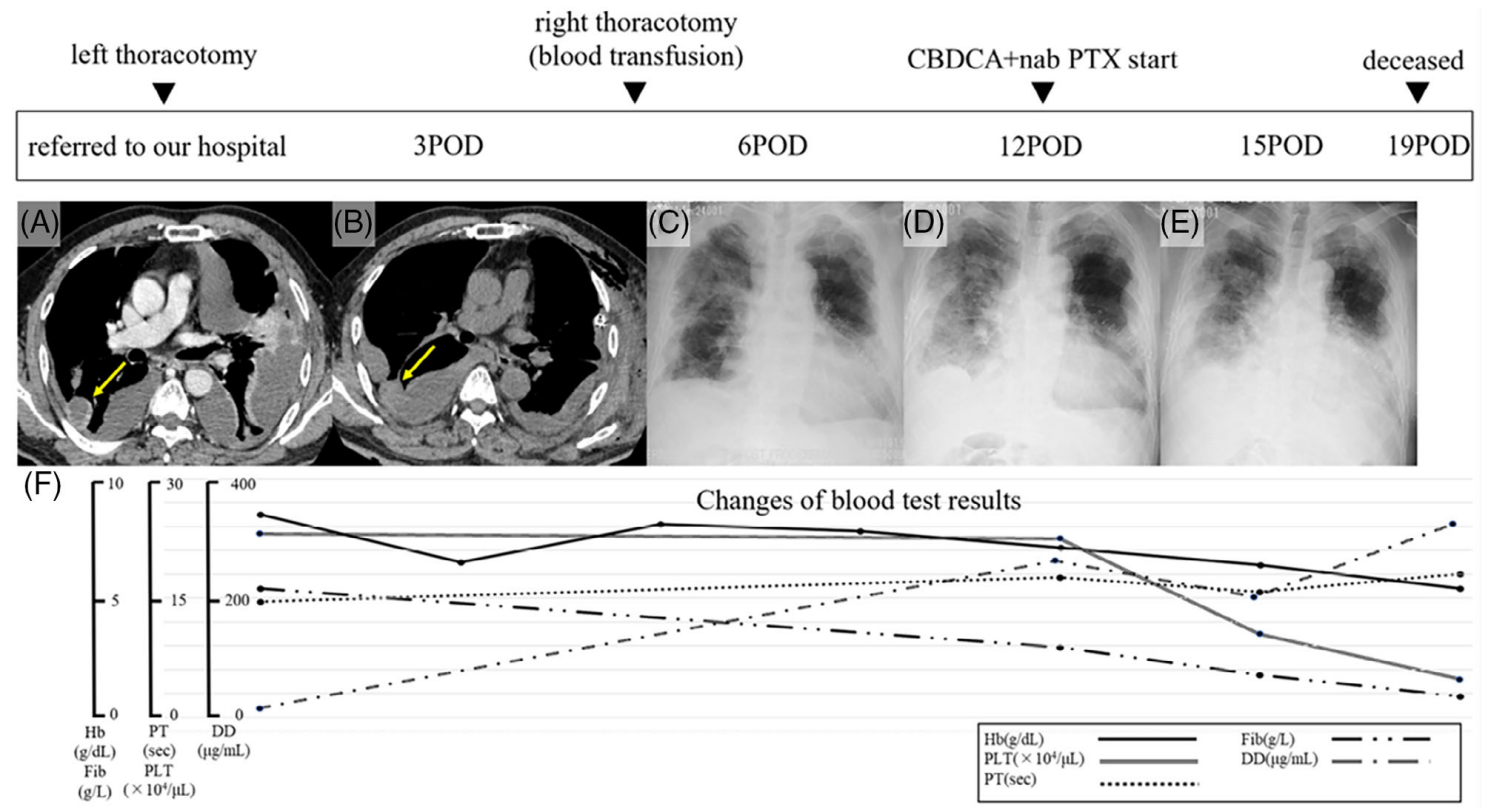
Clinical course, radiological image, and blood test results changes of the patient. (A) Bilateral pleural effusion of relatively high density was found. The amount of the left effusion was massive. (B) The amount of right effusion increased. The nodule of blood density was found (yellow arrow). (C) Chest x‐ray soon after the second operation. (D) The amount of pleural effusion started to increased again on postoperative day 12. (E) Increasing of pleural effusion continued (postoperative day 15). (F) Changes of the blood test results. Haemoglobin, platelet, and plasma fibrinogen level count decreased and prothrombin time and D‐dimer increased, especially from postoperative day 12. CBDCA, carboplatin; DD, D‐dimer; Fib, fibrinogen; Hb, haemoglobin; PLT, platelet; POD, postoperative day; PT, prothrombin time; PTX, paclitaxel.

A 72‐year‐old male patient with bilateral pleural effusions was transferred to our hospital by ambulance from a local hospital. He had a history of hepatitis C (sustained virological response), and benign adrenal tumour. He used no antiplatelet or anticoagulant drugs. On arrival, his blood pressure was 140/81 mmHg, heart rate was 98 bpm, respiratory rate was 23 breaths/min, and oxygen saturation < 90% on oxygen administration at 2 L/min with a nonrebreather mask. Blood test results revealed haemoglobin 8.5 g/dL. Platelet count, protrombin time (Figure 1F), international normalized ratio, activated partial thromboplastin time, and liver and renal function were at normal level. Contrast‐enhanced computed tomography (CT) revealed a bilateral pleural effusion of relatively high density (Figure 1A). No obvious extravasation was observed. The amount of left effusion was massive and chest tube drainage was undertaken. More than 1500 mL of bloody pleural effusion was continuously drained from the chest tube. With relatively rapid heart rate, we thought that the patient was in pre‐shock condition caused by massive hemothorax. Therefore, we decided to do thoracotomy for left hemothorax to stop bleeding and investigate the cause of haemorrhage.

**FIGURE 2:**
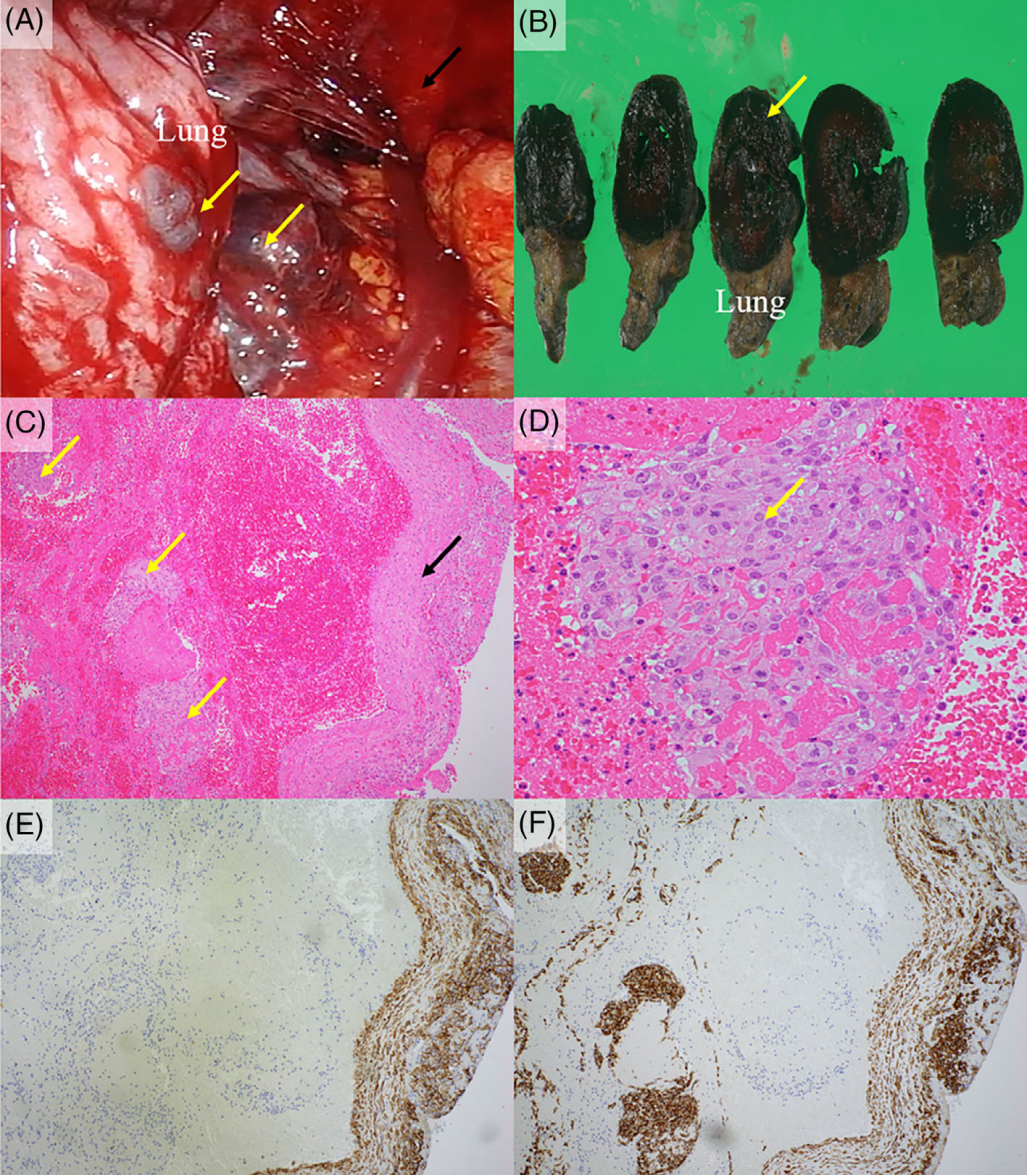
(A) Black coloured lesions which looks like hematoma (yellow arrow) adhere to the visceral pleura of the lung. The bleeding from the tumour was confirmed (black arrow). (B) The lesion was cystic (yellow arrow). (C) Microscopically, the cystic construction consisted of reactive mesothelial cells (black arrow), and inside the mesothelial cells, atypical cells with high nucleocytoplasmic (N/C) ratio mixed with hematoma was found (yellow arrows) (haematoxylin and eosin ×100). (D) Atypical cells with high nucleocytoplasmic (N/C) ratio (haematoxylin and eosin ×400). (E) Reactive mesothelial cells were positive for 2D‐40, but tumour cells negative. F: Both tumour cells and reactive mesothelial cells were positive for CK AE1/AE3.

Intraoperatively, we found various sizes of multiple lesions, which looked like hematoma, adhering to the visceral pleura of the lung (Figure 2A). The exudative bleeding from the lesions was confirmed. We considered the lesion as the cause of hemothorax and resected as many lesions as possible for haemostasis.

After the operation, the pleural effusion of the right thoracic cavity gradually increased (Figure 1B) and haemoglobin of the blood test decreased to 6.5 g/dL on postoperative day 3. The CT revealed nodules of blood density on the right lung (Figure 1A, B, yellow arrow), therefore, the operation for haemostasis was performed for right hemothorax (with intraoperative blood transfusion). The intraoperative finding was the same as those on the left side, and we resected as many hematoma‐like lesions as possible.

The histopathological diagnosis of the lesion was confirmed on postoperative day 11. It was a cystic lesion adhering to the visceral pleura (Figure 2B). Microscopically, the cystic construction consisted of reactive mesothelial cells, and inside the mesothelial cells, atypical cells with high nucleocytoplasmic (N/C) ratio mixed with hematoma were found (Figure 2C–F). Immunohistochemical staining showed that the atypical cells were positive for CK AE1/AE3 (Figure 2F), CK‐7, and Ki‐67 at 20%–30%, and negative for D2‐40, Calretinin, WT‐1, CEA, TTF‐1, NapsinA, p40, CK5/6, CK20, CDX‐2, PAX8, GATA3 and PSA. From these findings, the tumour was diagnosed as carcinoma, but no primary site was identified. Preoperative chest and abdomen contrast‐enhanced CT was rechecked to investigate the primary site of the tumour, but no apparent sign was found. Based on these findings, the tumour was diagnosed as carcinoma of unknown primary.

In spite of administration of carboplatin and nab‐paclitaxel from postoperative day 12, the amount of pleural effusion started to increased again (Figure 1C, D) and haemoglobin decreased again to 6.4 g/dL on postoperative day 15. Increasing of pleural effusion continued (Figure 1E) and on postoperative day 19, the bloody effusion started to ooze from the bilateral wound. Blood test revealed the further decrease of haemoglobin to 5.3 g/dL, and disseminated intravascular coagulation (DIC) (DIC score^2^; Platelet count 48 × 10^9^/L: 2, Prolongation of prothrombin time 3.7 s: 1, Plasma fibrinogen 0.87 g/L:1, D‐dimer level 324.7 μg/mL:2, total score 6 = overt DIC) (Figure 1F). We assessed that it is difficult to stop the bleeding with multiple tumours remaining condition. We decided not to perform further attempts at curative treatments. The bloody effusion continued to ooze from the wound and finally, the patient deceased from hemorrhagic shock.

## DISCUSSION

Spontaneous hemothorax is sometimes caused by neoplasia, and among neoplasia, angiosarcoma, schwannoma, thymoma, hepatocarcinoma, vascular tumours, soft tissue tumours, germ cell tumours, lung cancer, and mesothelioma have been reported as a cause.^1^ Hemothorax caused by carcinoma of unknown primary is very rare and to the best of our knowledge, this is the first report.

Surgical treatment for massive hemothorax is generally recommended in case of hemodynamic instability and evacuation of over 1500 mL through the chest tube and more than 200 mL/h blood loss through the chest tube over the first hour.^3^ In our case, we decided to perform a left thoracotomy in accordance with these recommendations. Retrospectively, the hemothorax was caused by oozing bloody effusion of the tumours and the condition was not an acute one. However, we could identify the cause of hemothorax from the finding of thoracotomy, and also, we believe that temporary haemostasis due to our bilateral thoracotomy elongated the duration of survival and helped to make time for getting a pathological diagnosis of the tumours.

Carcinoma of unknown primary is generally treated with regimens comprising platinum salts, and the prognosis have been reported to be very poor (median overall survival is generally <1 year) except for 10%–15% favourable‐risk subsets.^4^, ^5^ In our case, we administered carboplatin and nab‐paclitaxel, however, the patient deceased soon after the administration with the rapid progress of the disease.

In conclusion, we have experienced a very rare bilateral hemothorax caused by carcinoma of unknown primary. Because no other such cases have been reported in the past, it is difficult to plan the treatment. Further experience should be reported.

## CONFLICT OF INTEREST STATEMENT

None declared.

## ETHICS STATEMENT

The authors declare that appropriate written informed consent was obtained for the publication of this manuscript and accompanying images.

## Data Availability

The data underlying this article cannot be shared publicly for protecting privacy of individuals that participated in this study. The data may be shared on reasonable request to the corresponding author after an additional approval by the Institutional Review Board of Japanese Red Cross Society Nagano Hospital, Nagano, Japan.
